# Intermediate-Dose Tinzaparin Versus Low-Dose Enoxaparin as Thromboprophylaxis in Hospitalized Internal Medicine Patients

**DOI:** 10.3390/medsci14040425

**Published:** 2026-07-24

**Authors:** Aimilios Kaklamanos, Vasileios Serepisios, Sofia Chelioti, Kalliopi Zervakou, George Christodoulou, Abraham Pouliakis, Konstantina Mikelopoulou, Daphnie Germanou, Ilias Tsinokou, Dimitrios Dorotheos Papadakis, Christos Tsironis, Theodoros Androutsakos

**Affiliations:** 1 Department of Pathophysiology, School of Medicine, National and Kapodistrian University of Athens, 11527 Athens, Greece; emilioskaklamanos@gmail.com (A.K.); vasilis.sere@gmail.com (V.S.); sofiachelioti@gmail.com (S.C.); kalliazervakos@gmail.com (K.Z.); gvchristod@gmail.com (G.C.); nadiamikelopoulou123@gmail.com (K.M.); dafniegermanou@gmail.com (D.G.); ilcinok@med.uoa.gr (I.T.); dimitriosdorotheospapadakis@gmail.com (D.D.P.); tsironisc@med.uoa.gr (C.T.); 2 2nd Department of Pathology, University Hospital “Attikon”, National and Kapodistrian University of Athens, 12461 Athens, Greece; apouliak@med.uoa.gr

**Keywords:** tinzaparin, enoxaparin, thrombosis prophylaxis, bleeding

## Abstract

**Background/Objectives**: Acutely ill patients in hospital are at high risk for thrombosis, even post-discharge. However, the safest and most effective strategy for thromboprophylaxis in such patients is still under debate. Our aim was to compare the subcutaneous use of low-dose prophylactic enoxaparin versus intermediate-dose tinzaparin. **Methods**: Patients in an internal medicine clinic of a tertiary hospital in Athens, Greece, with increased risk for thrombosis (PADUA score ≥ 4) received either intermediate-dose tinzaparin (8000 anti-Xa units) or 40 mg enoxaparin as thromboprophylaxis. Efficacy and adverse effects were evaluated and compared up to 14 days post-discharge. **Results**: A total of 251 patients who received tinzaparin were compared with 95 patients under enoxaparin thromboprophylaxis. No statistically significant difference was found in the number of bleeding and/or clinically evident thrombotic events between the two groups [6 versus 14 bleeding (*p* = 0.2603) events and 0 versus 1 venous thrombotic event (*p* = 0.999) in the enoxaparin and tinzaparin groups, respectively]. Moreover, no difference was found in length of stay [enoxaparin: 10 days (95%CI: 9–12 days); tinzaparin: 8 days (95%CI: 8–10 days, HR = 1.18, 95%CI: 0.91–1.54, *p* = 0.2] or mortality [11 deaths with enoxaparin (11.58%, 95%CI: 6.59–19.55%) and 50 deaths with tinzaparin (19.92%, 95%CI: 15.45–25.30%, OR: 1.9, 95%CI: 0.94–3.83, *p* = 0.069)]. The use of thromboprophylaxis for two weeks post-discharge did not result in any bleeding or clinically evident thrombotic events in either group. **Conclusions**: In our cohort, the use of intermediate-dose tinzaparin was similar to the use of low-dose enoxaparin for patients with a high PADUA score in terms of thrombotic and/or bleeding events.

## 1. Introduction

Venous thromboembolism (VTE) is the third most common cardiovascular diagnosis, with a yearly incidence exceeding 1 in 1000 for white Europeans, reaching up to 1% in the elderly. Up to 50% of all VTE cases appear during a hospitalization or up to 3 months post-discharge and are associated with increased morbidity and mortality [1,2,3,4].

Various hospitalization-associated risk factors have been identified for VTE, including, but not limited to, acute medical disease, surgery, trauma, immobilization, malignancy, cancer treatment, heart failure, respiratory failure, thrombophilia, intravenous catheter use, previous VTE, obesity and increased age [5]. For the assessment of such risk factors and the identification of patients who may benefit most from antithrombotic modalities, different scores have been developed [6]. One of the most commonly used is the PADUA risk assessment score (Table 1). Patients with a PADUA score of ≥4 are considered high-risk and should therefore receive prophylactic antithrombotic therapy [7].

Historically, in patients who have a high risk for VTE, various approaches have been applied, including anticoagulants, mechanical measures and inferior vena cava filters. Current guidelines suggest the use of pharmacological thromboprophylaxis as first-line prophylaxis, while mechanical measures are only suggested in patients already on anticoagulants as an adjunctive measure or in patients with very high bleeding risk in whom pharmacological anticoagulation is contraindicated. As first-line anticoagulation, most guidelines suggest the use of parenteral low-molecular-weight heparins (LMWH) like enoxaparin, tinzaparin and fondaparinux. Direct oral anticoagulants (DOACs) (rivaroxaban, apixaban, etc.) or vitamin K antagonists have been administered as second-line agents but showed increased risk for bleeding and are therefore not widely indicated [8,9,10,11]. Although it has been reported that thromboprophylaxis discontinuation upon hospital discharge results in higher VTE incidence [12], existing guidelines are contradictory with respect to whether thromboprophylaxis should be continued upon discharge or not [11,13].

Even though the benefit of thromboprophylaxis is well established, the dose is still under debate [14,15,16,17]. Most guidelines suggest the use of fixed-dose LMWH, such as 40 mg enoxaparin or 4500 anti-Xa units of tinzaparin, unless patients are severely obese or have impaired renal function [18,19,20]. On the other hand, the use of these fixed-dose regimens could lead to increased bleeding tendency in underweight patients [21,22,23].

Moreover, recent studies in acutely ill patients have shown mixed results regarding the use of different doses of tinzaparin as thromboprophylaxis [24,25]. Therefore, the goal of this study was to compare the use of intermediate doses of tinzaparin and the use of prophylactic fixed-dose enoxaparin as standard of care in high-risk internal medicine patients (defined as those with PADUA score of ≥4) [Table 1].

## 2. Materials and Methods

### 2.1. Study Design

This is an observational, prospective, study, comparing the use of intermediate-dose tinzaparin as thromboprophylaxis versus low-dose enoxaparin as thromboprophylaxis in terms of both efficacy and safety. The study cohort includes all eligible patients hospitalized in an internal medicine department from 1 December 2023 to 1 October 2025, receiving tinzaparin 8000 anti-Xa during their hospitalization and for 14 days after hospital discharge. The administered dose followed a one-size-fits-all regimen, namely, 65% of the therapeutic dose for an adult of ~70 kg. The intermediate dose of tinzaparin that was used in various studies varies from 50% to 75% of the therapeutic dose. In our institution, a fixed-dose strategy is preferred over individualized weight-based dosing in order to simplify drug administration since it corresponds to a dose of around 100 IU/kg for most patients—a common intermediate regimen [25,26]. All patients were followed up from hospitalization day 1 up to 2 weeks post-discharge. The final follow-up was performed via a phone patient assessment. Patients with a decreased glomerular filtration rate (GFR) (<30 mL/min/1.73 m^2^) or body weight < 50 kg received 4500 anti-Xa units tinzaparin. To minimize possible biases, the treating clinicians did not participate in data collection and analysis. The control group included patients receiving 40 mg of enoxaparin subcutaneously (or 20 mg when their GFR was below 30 mL/min/1.73 m^2^ or their body weight was below 50 kg). The enrolled patients fulfilled all the inclusion criteria during the study period. No specific matching was performed since the study was designed to be as minimally interventional as possible. The patients’ data analysis showed that both study groups had similar baseline demographic and clinical characteristics, including age, sex and major comorbidities (as shown in Table 2).

Anti-Xa activity for the LMWH activity/efficacy monitoring was not measured as it is not the routine clinical practice in our institution. Moreover, the use of anti-Xa activity is not routinely suggested/performed in the literature [27,28]. Age, gender and co-morbidities were matched between the two groups (Table 2).

Inclusion criteria:

1. Patients admitted to the internal medicine ward suffering from an acute medical disease and with a PADUA score ≥ 4. *
2. Age ≥ 18.
3. Signed informed consent.
4. No active bleeding. **

Exclusion criteria ***:

1. Patients with a PADUA score < 4.
2. Age < 18.
3. Pregnancy.
4. Current or previous diagnosis or suspicion of pulmonary embolism (PE) or deep vein thrombosis (DVT) or any other condition necessitating the use of therapeutic anticoagulation upon hospital admission or during hospitalization (e.g., atrial fibrillation). ^#^

* Patients admitted with a lower PADUA score but who were bed-ridden for more than 3 days, anticoagulation was administered later on, i.e., when the required PADUA score was reached.

** Patients admitted due to active bleeding, anticoagulants were initiated only in the event that the bleeding was fully resolved, they had a PADUA score ≥ 4, and the administration of anticoagulants was considered safe in their clinical condition.

*** Previous anticoagulation therapy (even if it was with an LMWH) was not an exclusion criterion if the use was not for therapeutic purposes.

# Patients with current thrombosis or DVT who did not need therapeutic anticoagulation (due to other treatments, other comorbidities, etc.), and who fulfilled the rest of the inclusion criteria, were also included.

Documentation

The following data were collected for each patient:

- Demographic characteristics [sex, date of birth, body weight, height, body-mass index (BMI), etc.].
- Medical history (past thrombotic and bleeding events, risk factors, recent surgery, comorbidities, chronic anticoagulation, etc.).
- Characteristics of the disease that led to the patient’s hospitalization.
- PADUA score.
- Administered thromboprophylaxis.
- Bleeding events or clinically evident thrombotic events (DVT or PE) during hospitalization and up to 2 weeks post-discharge.
- Other side-effects associated with the use of thromboprophylaxis.
- Duration of hospitalization.

### 2.2. Goals of the Study

The primary goal of this study was to compare the efficacy and safety of intermediate-dose tinzaparin with fixed-dose enoxaparin as thromboprophylaxis during hospitalization and for two weeks post-discharge in high-VTE-risk internal medicine patients hospitalized for an acute medical disease.

### 2.3. Definitions

(1) Thrombotic events

Clinically evident thrombotic events were documented/confirmed by imaging studies such as triplex ultrasonography or CT angiography. These included DVT, PE and splanchnic venous thrombosis.

(2) Hemorrhagic events were defined according to the International Society of Thrombosis and Haemostasis as major bleeding, clinically relevant non-major bleeding (CRNMB), and minor bleeding [29,30]:
  - Major bleeding was defined as evident blood loss followed by a hemoglobin decrease of 2 g/dL or more, blood loss that leads to a blood transfusion of ≥2 blood units, blood loss that occurs within a vital organ (retroperitoneal, intracranial, intraorbital, intraspinal, intra-articular, pericardial, intramuscular followed by compartment syndrome), or blood loss that contributes to death.
  - CRNMB was defined as evident blood loss that does not fulfill the criteria for major bleeding but needs medical intervention, scheduled medical examination, or (temporary) antithrombotic therapy discontinuation, or is not tolerated by the patient (e.g., due to pain or limitation of daily activities).
  - All other bleeding events were categorized as minor.

### 2.4. Statistical Analysis

The statistical analysis was performed using the R language (R Foundation for Statistical Computing, Vienna, Austria) version 4.5.1. No a priori sample size calculation was performed, as patient recruitment was based on consecutive eligible patient inclusion during the study period. Enrollment was completed according to the recruitment period. We acknowledge that the absence of formal sample size estimation represents a limitation of this study. Thus, we have provided the 95%CI and SD for the categorical and numeric data, respectively, as a measure of uncertainty. Descriptive characteristics for the quantitative data were expressed as median and range and, for completeness reasons, via mean ± standard deviation (SD). For the qualitative data, we reported the frequency of occurrence and the relevant percentage. For the qualitative parameters, statistical tests were performed via the chi-square test (and, if required, a Fisher exact test), and for the arithmetic data (when normality was not confirmed using the Shapiro–Wilk test), non-parametric tests were applied, specifically the Mann–Whitney U test. When data normality within the classes under comparison was confirmed, the *t*-test was applied. Furthermore, to identify potential differences in the percentage of thrombotic events and bleeding events, we applied the z-test for proportions. The significance level (*p*-value) was set to 0.05, and when applicable, tests were two sided. In terms of length of stay, the relevant Kaplan–Meier plots were considered and were compared using the log-rank method. Length of stay was defined as the period from the admission date to the discharge date. Events were considered when a patient exited alive from the clinic. No formal sample size calculation was performed prior to the study, and due to the absence of statistically significant findings, post hoc power calculations were not conducted as they provide limited additional information. Instead, effect estimates are presented alongside their corresponding 95% confidence intervals to reflect the precision and uncertainty of the study findings. To account for potential confounding factors and to adjust accordingly, we applied multivariable logistic regression; specifically, variables entered into the model had *p* < 0.1 in the univariable analysis. Furthermore the various comorbidities were not used as individual predictors; instead, the Charlson comorbidity index (CCI) was used as a single comorbidity marker; individual comorbidities were too numerous and would make the model too complex; additionally, they were dependent on CCI, making the model potentially unstable. No other clinically relevant covariates were forced as inputs and no imputation or other special handling of missing data was performed. In addition, gender was initially not involved in the analysis; however, it proved significant in the univariable analysis and was thus included in the multivariable analysis, where it was eventually found to be non-significant. The relevant descriptions have changed and the results table has changed. In addition, we added the univariable analysis results into the table to provide additional details. No changes in the conclusions were found. Statistical analyses were conducted on available data for each comparison. In logistic regression models, observations with missing values in any predictor or outcome variable were excluded from the analysis (complete-case analysis).

### 2.5. Ethical Approval

The study was conducted in accordance with the Declaration of Helsinki, and was approved by the Laiko General Hospital Ethics Committee (Approval No. 17776/27-11-2023) on 27 November 2023.

## 3. Results

Overall, 251 patients who received intermediate-dose tinzaparin were compared to 95 patients who received low-dose enoxaparin as thromboprophylaxis. The two groups did not differ significantly in their demographic and medical characteristics, as shown in Table 2. The various causes of hospital admission for these patients are depicted in Supplementary Table S1; most patients were admitted due to infectious or gastrointestinal diseases, with no differences between the two groups. The most common factors that led to an increased PADUA score in either group were reduced mobility (294 patients), advanced age (273 patients), and acute infection and/or rheumatologic disorder (234 patients). Interestingly, none of the patients reported a thrombophilic condition; however, it is important to clarify that we did not test all these patients for thrombophilia, but we did evaluate their medical history for known thrombophilic conditions. In addition, 5–10% of the patients had suffered a recent trauma or undergone surgery. From these patients, the vast majority (15 in the tinzaparin group and 3 in the enoxaparin group) had undergone hip replacement surgery and were on prophylactic anticoagulation with low-dose LMWH. Two more patients from the tinzaparin group had undergone laparoscopic cholecystectomy. The rest had undergone surgery due to a fall-related limb fracture.

The use of intermediate doses of tinzaparin as thromboprophylaxis did not differ significantly from 40 mg of enoxaparin in terms of thrombosis prevention and safety (Table 3). Only one patient suffered a clinically evident thrombotic event during tinzaparin treatment (DVT). On the other hand, no patients were found to have suffered a clinically evident thrombosis during enoxaparin treatment. Supplementary Table S2 depicts various possible risk factors for thrombosis and/or bleeding; however, due to the low number of thrombotic events, no statistical correlations were possible. In addition, 14 and 6 patients had a bleeding episode under tinzaparin and enoxaparin use, respectively (Table 4 and Table 5). The majority of these bleeding events occurred in the gastrointestinal (GI) and urinary tracts and were of minor significance. Interestingly, no bleeding recurrence was seen after thromboprophylaxis re-initiation following the acute bleeding phase. A total of six patients, five under tinzaparin and one under enoxaparin treatment, suffered a major bleeding episode leading to thromboprophylaxis cessation. From these patients, one from the enoxaparin group and three from the tinzaparin group died due to aspiration pneumonia and/or nosocomial infection and sepsis. Interestingly, the use of either tinzaparin or enoxaparin for 2 weeks post-discharge was not associated with any thrombotic or bleeding events.

In terms of mortality, no statistically significant difference was found between the two groups. From the 95 patients that received enoxaparin, 11 died (11.58%) [95%CI: 6.59–19.55%], while from the 251 patients treated with tinzaparin, 50 died (19.92%) [95%CI: 15.45–25.30%], OR: 1.9 [95%CI: 0.943–3.828], *p* = 0.069. None of these deaths could be directly attributed to the use of either antithrombotic therapy. A multivariable logistic regression analysis was performed to investigate the association between clinical and demographic factors and survival. The model included patient survival (aiming to predict survival probability) as a dependent variable, and the treatment group, age, gender, weight, BMI, PADUA score, and CCI were included as input variables (resulting from the univariable analysis). As shown in Table 6, only the PADUA score was significantly associated with the outcome (OR = 0.75, 95%CI: 0.61–0.93, *p* = 0.007), indicating that higher PADUA scores were associated with lower odds of survival in this model. Treatment group, age, gender, weight, BMI, and Charlson comorbidity index (CCI) were not significantly associated with mortality (all *p* > 0.05). The area under the curve of the multivariable model was 0.715.

**Figure 1:**
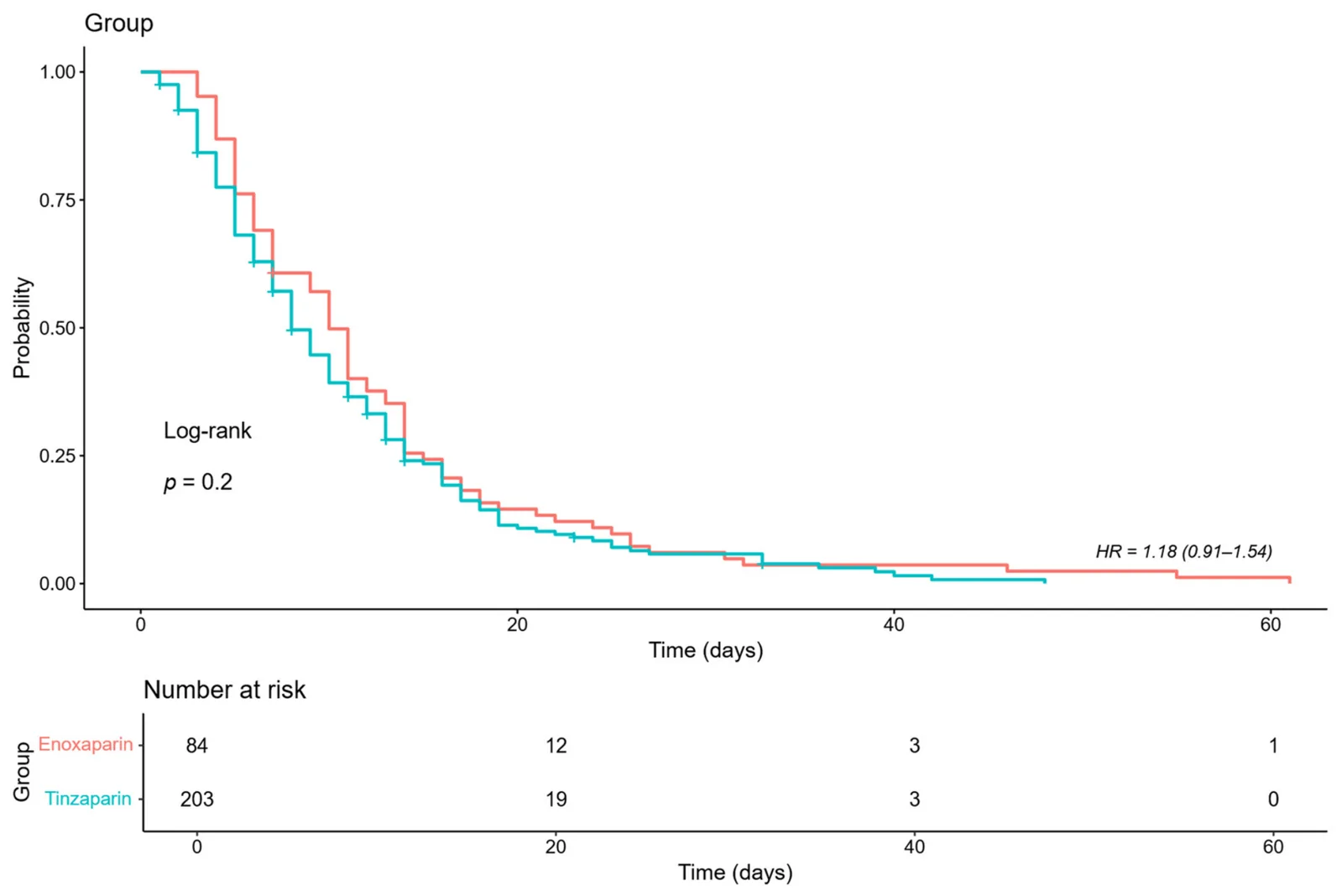
Kaplan–Meier curves for patient length of stay for each study group.

Moreover, the use of either agent was not associated with a difference in the length of stay [the median length of stay for enoxaparin was 10 days (95%CI: 9–12 days), and the median length of stay for tinzaparin was 8 days (95%CI: 8–10 days, HR = 1.18, 95%CI: 0.91–1.54, *p* = 0.2)]. As shown in Figure 1, Kaplan–Meier survival analysis showed no statistically significant difference in duration of hospitalization between patients treated with enoxaparin or tinzaparin during follow-up (*p* = 0.20). The estimated hazard ratio was 1.18 (95%CI: 0.91–1.54), suggesting no significant association between treatment group and duration of hospitalization. Kaplan–Meier curves show overlap throughout the observation period, without clear separation favoring either treatment strategy. The number of patients at risk progressively declined over time in both groups. Finally, no other significant side-effects were documented in either group apart from known local irritation or bruising, although two of the patients in the tinzaparin group suffered an ischemic stroke during hospitalization.

## 4. Discussion

LMWH (tinzaparin, enoxaparin, dalteparin, etc.) have long been shown to be effective as thrombosis prevention regimens in the majority of patients [31]. For example, the MEDENOX and PRINCE [32,33], the PREVENT and PROTECT [34,35], and the ARTEMIS studies established the role of enoxaparin, dalteparin, and fondaparinux, respectively, as thromboprophylaxis in critically ill medical patients. On the other hand, tinzaparin seems to have some additional features in comparison to other LMWH, namely, anti-inflammatory properties and non-renal excretion [36]. For these reasons, tinzaparin has been widely studied in various specific populations such as patients with neoplasia, renal impairment, and COVID-19 [37,38].

However, despite the proven benefit of anticoagulation using LMWH in medical patients, the effect of these drugs in terms of length of hospitalization, final outcome, etc., is yet to be determined [39]. For example, the LIFENOX trial found no difference in the mortality of patients receiving enoxaparin, although thrombosis prevention was achieved without any severe bleeding risk [40]. In addition, the safest and most effective strategy of LMWH usage (one-size-fits-all versus a more personalized approach) is still under debate, even though most current data seem to support the use of a more personalized approach [41]. Finally, the duration of anticoagulation has also been a matter of debate in the literature, since thrombotic risk seems to be increased in medical patients even after their hospital discharge. For example, the EXCLAIM study showed that prolonged enoxaparin treatment is associated with reduced thrombotic events even after the mobilization and discharge of the patient [42]. On the contrary, the SYMPTOMS trial did not find any difference in the development of symptomatic DVT at 30 days upon discharge [43].

As for tinzaparin, various dosage regimens have been studied and compared with each other, although the results are still contradictory. For example, the INTERACT study showed a protective advantage of increased doses of tinzaparin in COVID-19 patients without an increase in bleeding risk [37]. On the contrary, the PROTHROMCOVID study did not find any difference in the efficacy of various tinzaparin dosages, while it showed an increased bleeding risk with increased tinzaparin dose in COVID-19 patients [25]. In addition, another study on non-COVID-19 critically ill medical patients found a benefit from the use of intermediate-dose (versus low-dose) tinzaparin in terms of duration of hospitalization and mortality [24].

Our prospective observational study tried to compare the efficacy and safety of low-dose enoxaparin versus intermediate-dose tinzaparin in critically ill medical patients with increased thrombotic risk (according to the PADUA score). In our cohort of 346 patients hospitalized in an internal medicine clinic of a tertiary hospital in Greece, the use of either 40 mg enoxaparin or 8000 anti-Xa units of tinzaparin was not found to have a statistically significant effect regarding the incidence of either thrombotic or bleeding events. Bleeding occurred in a total of 20 patients (14 under tinzaparin and 6 under enoxaparin treatment), while thrombotic episodes were extremely rare, with only 1 patient having clinically evident venous thrombosis (DVT). Although it is known that antithrombin levels below 60% are associated with decreased LMWH efficacy, we did not measure antithrombin levels in our cohort since we did not have any indications of possible LMWH resistance [44]. Both regimens showed no statistically significant difference regarding side-effects, although two patients that were receiving tinzaparin suffered an ischemic stroke. In addition, no difference was found between the two agents in either patient mortality or length of stay. Although mortality was higher in the tinzaparin group, it did not reach statistical significance. In addition, none of the deaths could be directly associated with the use of any of the two medications. Finally, the use of either enoxaparin or tinzaparin two weeks post-discharge was associated with no thrombotic or bleeding events in both groups.

The above findings, despite the low power of our study, agree with the findings from other studies that show that intermediate-dose tinzaparin use is associated with protection against thrombosis without introducing any major bleeding risk [24,37]. However, we did not find any favorable effects of the use of intermediate-dose tinzaparin in terms of treatment duration, length of stay, and mortality, as was the case in the paper by Akinsoglou et al. [24].

Our study has some severe limitations. First, it was performed in an internal medicine clinic in Athens, including mainly Caucasian patients with no recent surgeries, so our results cannot be generalized to all patients. Second, it was a non-randomized, non-blind observational study, so the risk of bias cannot be totally excluded. Moreover, since treatment allocation was based on physician judgment rather than randomization, residual confounding by indication cannot be completely excluded despite the fact that both study groups had similar baseline demographic and clinical characteristics and despite multivariable adjustment. Third, no triplex ultrasonography was performed in all patients, so possible clinically silent DVTs were not detected. This potential underestimation of thrombotic events could further negatively affect the statistical power of our analysis. However, this study aimed to follow everyday clinical practice, where triplex angiography is performed only when thrombosis is suspected. The relatively small number of events, imbalance in group sizes, and limited statistical power limit the precision of our estimates and do not allow us to reach a firm conclusion. In addition, the short follow-up of the patients after their discharge may also affect the results of our study, since it has been shown that DVT and/or PE can occur even at 90 days post-discharge [45,46,47]. Finally, although no difference was found between the two groups in terms of mortality, the *p* value was marginally non-significant, which suggests that a similar study with larger patient groups is needed to clarify our findings.

## 5. Conclusions

Despite the above-mentioned limitations, we have shown that in our cohort, the use of intermediate doses of tinzaparin as thromboprophylaxis in patients with an increased PADUA score did not result in improvement of thrombotic or an increase in bleeding events when compared with low-dose prophylactic enoxaparin. Moreover, the use of either agent is not associated with increased length of stay, which is a known predictive factor for complications and in-hospital death. Finally, the use of intermediate-dose tinzaparin or low-dose enoxaparin in a prolonged regimen for 2 weeks post-discharge did not result in any thrombotic or bleeding events. However, more studies with more statistical power and larger group sizes are needed in order to reach firm conclusions about the comparison of these two antithrombotic regimens.

## Figures and Tables

**Table 1:** The PADUA risk assessment score.

Risk Factors	Score
Immobilization *	3
Active malignancy **	3
Previous episode of VTE (apart from superficial thrombophlebitis)	3
Known thrombophilia ***	3
Recent (≤1 month) trauma or surgery	2
Advanced age (≥70)	1
Heart or respiratory failure	1
Acute myocardial infarction or ischemic stroke	1
Hormonal therapy	1
Obesity (BMΙ > 30)	1
Acute infection and/or rheumatologic disease	1

* Patient is able or allowed to get off the bed only to go to the toilet for at least 3 days. ** Patients with local or distant metastases and/or recent chemotherapy or radiotherapy (during the past 6 months). *** Carriers of decreased or absent natural coagulation inhibitors (antithrombin, protein C, protein S), factor V Leiden, prothrombin mutation G20210A, or antiphospholipid syndrome.

**Table 2:** The basic demographic and medical characteristics of the patients who received either enoxaparin or tinzaparin. No statistically significant difference was found between the two patient groups.

-	Enoxaparin (N = 95)	Tinzaparin (N = 251)	*p* Value Adjusted
Age	-	-	-
Mean ± SD	76.68 ± 15.35	79.49 ± 13.28	0.252
Median [Q1, Q3]	80.00 [70.50, 88.00]	83.00 [72.00, 89.00]	-
Gender (male)	51 (53.68%) [43.71–63.37%]	103 (41.04%) [35.13–47.21%]	0.165
BMI (Kg/m^2^)	-	-	-
Mean ± SD	24.55 ± 3.786	25.46 ± 5.023	0.138
Median [Q1, Q3]	23.53 [21.92, 26.26]	25.03 [23.10, 27.08]	-
History of thrombosis	17 (17.89%) [11.48–26.80%]	57 (22.80%) [18.03–28.39%]	0.456
Bleeding history	7 (7.45%) [3.65–14.58%]	15 (6.02%) [3.68–9.70%]	0.718
Smoker or ex-smoker	25 (26.32%) [18.51–35.97%]	94 (37.75%) [31.96–43.92%]	0.165
Varicose veins	10 (10.64%) [5.88–18.49%]	11 (4.40%) [2.47–7.71%]	0.165
Family history of thrombosis	0 (0.00%) [0.00–3.89%]	1 (0.40%) [0.07–2.24%]	1
Central venous catheter	4 (4.21%) [1.65–10.33%]	6 (2.40%) [1.10–5.14%]	0.621
Recent surgery	5 (5.26%) [2.27–11.73%]	21 (8.37%) [5.54–12.45%]	0.456
Cardiac disease (heart failure)	29 (30.53%) [22.17–40.39%]	56 (22.31%) [17.60–27.86%]	0.252
Arterial hypertension (not well controlled)	15 (15.79%) [9.81–24.43%]	17 (6.77%) [4.27–10.58%]	0.123
Diabetes mellitus	21 (22.11%) [14.94–31.44%]	71 (28.29%) [23.07–34.15%]	0.433
Renal insufficiency (GFR < 30 mL/min)	20 (21.05%) [14.06–30.29%]	31 (12.35%) [8.84–17.00%]	0.165
Liver failure	2 (2.11%) [0.58–7.35%]	0 (0.00%) [0.00–1.51%]	0.187
Inflammatory/autoimmune disease	8 (8.42%) [4.33–15.75%]	13 (5.18%) [3.05–8.66%]	0.433
Thyroid disease	22 (23.16%) [15.82–32.58%]	52 (20.72%) [16.16–26.15%]	0.718
Dyslipidemia	36 (37.89%) [28.79–47.94%]	70 (27.89%) [22.71–33.74%]	0.187
Respiratory disease	18 (18.95%) [12.33–27.97%]	40 (15.94%) [11.93–20.97%]	0.629
Use of LMWH	-	-	-
Fondaparinux	2 (66.67%) [20.77–93.85%]	1 (4.17%) [0.74–20.24%]	0.187
Enoxaparin	1 (33.33%) [6.15–79.23%]	16 (66.67%) [46.71–82.03%]	-
Tinzaparin	0 (0.00%) [0.00–56.15%]	5 (20.83%) [9.24–40.47%]	-
Bemiparin	0 (0.00%) [0.00–56.15%]	2 (8.33%) [2.32–25.85%]	-
Antiplatelet	19 (20.65%) [13.64–30.02%]	37 (14.92%) [11.02–19.89%]	0.395
Postmenopausal	40 (93.02%) [81.39–97.60%]	140 (97.22%) [93.08–98.91%]	0.3669
PADUA score	-	-	-
Mean ± SD	5.44 ± 1.39	5.59 ± 1.41	0.456
Median [Q1, Q3]	5 [4, 6]	5 [5, 6]	-
Charlson Comorbidity Index	-	-	-
Mean ± SD	5.9 ± 2.74	6.24 ± 2.60	0.2959
Median [Q1, Q3]	6 [4, 7.5]	6 [5, 8]	

**Table 3:** Comparison of bleeding and thrombotic events in medical patients with an increased PADUA score. The use of an intermediate dose of tinzaparin as thromboprophylaxis did not show any difference in terms of thrombotic and/or bleeding events in comparison to low-dose enoxaparin.

-	Enoxaparin (n = 95)	Tinzaparin (n = 251)	*p* Value
Thrombotic events	0 (0%)	1 (0.4%)	0.999 *
Bleeding events	6 (6.33%)	14 (5.58%)	0.799 *
Mortality	11 (11.58%)	50 (19.92%)	0.069 *

*: Fisher’s exact test.

**Table 4:** Bleeding events severity. The majority of bleeding events documented were of minor significance and led only to temporary interruption of the antithrombotic therapy.

Bleeding Severity	Enoxaparin (n = 95)	Tinzaparin (n = 251)	Total	*p* Value *
minor	3, 3.16% [1.08–8.88%]	6, 2.39% [1.1–5.12%]	9, 2.6% [1.37–4.87%]	0.689
CRNMB	1, 1.05% [0.19–5.72%]	3, 1.2% [0.41–3.45%]	4, 1.16% [0.45–2.93%]	0.912
major	2, 2.11% [0.58–7.35%]	5, 1.99% [0.85–4.58%]	7, 2.02% [0.98–4.12%]	0.944
Total	6, 6.32% [2.93–13.1%]	14, 5.58% [3.35–9.14%]	20, 5.78% [3.77–8.76%]	0.795

*: test for proportions.

**Table 5:** Bleeding events location. Most bleeding events occurred in the gastrointestinal and the urinary tract. PTC: percutaneous transhepatic cholangiostomy.

Site of Bleeding	Enoxaparin	Tinzaparin	Total
Urinary tract	3	5	8
PTC	1	0	1
Gastrointestinal tract	1	8	9
Intracranial	1	0	1
Intramuscular	0	1	1

**Table 6:** Uni- and multivariable analysis for patient survival. BMI: body-mass index; CCI: Charlson comorbidity index.

	Univariable Analysis *p*	Multivariable OR [95%CI]	Multivariable *p* Value
Group (ref: Tinzaparin)	0.070	0.55 [0.26–1.14]	0.109
Age	0.005	0.99 [0.96–1.02]	0.411
Gender (ref: Female)	0.081	0.5 [0.22–1.13]	0.094
Weight (kg)	0.007	0.99 [0.92–1.05]	0.682
BMI (Kg/m^2^)	0.021	1.13 [0.91–1.39]	0.274
PADUA score	0.002	0.75 [0.61–0.93]	0.007
CCI	<0.001	0.9 [0.8–1.02]	0.096

## Data Availability

The original contributions presented in this study are included in the article/Supplementary Material. Further inquiries can be directed to the corresponding author.

## Supplementary Materials

The following supporting information can be downloaded at: https://www.mdpi.com/article/10.3390/medsci14040425/s1, Table S1: Causes of admission in the internal medicine ward for the patients that were treated either with enoxaparin or with tinzaparin; Table S2: Frequency of possible risk factors for DVT in patients taking antithrombotic therapy. Unfortunately, due to the rarity of the thrombotic incidents, no factor could be statistically associated with thrombosis. DOAC: direct oral anticoagulant.

## Abbreviations

The following abbreviations are used in this manuscript:

BMI	Body-mass index
CCI	Charlson comorbidity index
CI	Confidence interval
CT	Computed tomography
CRNMB	Clinically relevant non-major bleeding
DOACs	Direct oral anticoagulants
DVT	Deep vein thrombosis
GFR	Glomerular filtration rate
GI	Gastrointestinal
HR	Hazard ratio
LMWH	Low-molecular-weight heparins
OR	Odds ratio
PE	Pulmonary embolism
PTC	Percutaneous transhepatic cholangiostomy
UTI	Urinary tract infection
VTE	Venous thromboembolism
