# Supplementary material for: Intermediate-Dose Tinzaparin Versus Low-Dose Enoxaparin as Thromboprophylaxis in Hospitalized Internal Medicine Patients
Source: Med Sci (Basel). 2026 Jul 24;14(4):425. doi: 10.3390/medsci14040425 (PMC13515995; doi:10.3390/medsci14040425)
Supplement: Supplementary file 1 [file medsci-14-00425-s001.zip › medsci-4402468-supplementary.pdf]

Supplementary material

Table S1. Causes of admission in the internal medicine ward for the patients that were treated either with enoxaparin or with tinzaparin.

	Reason for Admission	Enoxaparin	Tinzaparin
Infection (183)	COVID-19 infection	1	4
	Sepsis	1	3
	Microbemia	4	10
	Respiratory infection	17	26
	Spondylodiscitis	0	1
	Septic shock	5	6
	Urinary tract infection	2	25
	Fever	5	17
	Osteomyelitis	1	1
	Soft tissue infection	5	18
	Aspiration pneumonia	1	14
	Pyelonephritis	1	2
	Post-operation infection	0	1
	Infective endocarditis	1	0
	Clostridium difficile enterocolitis	1	1
	Spontaneous bacterial peritonitis	1	0
	Lymphadenopathy and fever	0	1
	Paravertebrate abscess	0	1
	Influenza A	1	1
	Liver abscess	0	1
	Urine tract infection—pneumonia	0	2
	Respiratory infection- heart failure dysregulation	1	0
Gastrointestinal (53)	Jaundice	2	2
	Cholelithiasis	1	1
	Primary sclerosing cholangitis	1	0
	Dysphagia due to esophageal surgery	1	0
	Colitis	1	0
	Diverticulitis	1	2
	Constipation	0	1
	Cholangitis	1	8
	Acute pancreatitis	0	2
	Abdominal pain, diarrhea, vomiting	3	5
	Cholecystitis	1	5
	Ileus	2	3
	Hepatic encephalopathy	0	1
	Recurrent pancreatitis	0	1
	Upper gastrointestinal bleeding	2	2
	Hematochezia	2	0
	Scheduled patient admission for PTC	1	0
	Gastrostomy blockade	1	0
Cardiovascular (21)	Shortness of breath due to aortic stenosis	1	0
	Vagotonic episode	1	1
	Loss of sensation, possible stroke	0	1
	Ischemic stroke	4	4
	Lactic acidosis from metformin	0	2

	Deep vein thrombosis	0	1
	Lower limb thrombosis and thrombectomy	1	0
	Heart failure deregulation	0	2
	Pre-syncopal episode	0	1
	Lymphedema	1	1
Hematological (17)	Anemia*	3	8
	Sickle cell anemia—acute chest syndrome	0	2
	Polycythemia vera	0	1
	Lymphoma recurrence	1	0
	Admission due to scheduled chemotherapy for NK T cell Lymphoma	0	1
	Idiopathic Thrombocytopenic Purpura (ITP) **	0	1
Pulmonary (19)	Type 2 respiratory failure	2	6
	Dyspnea	1	7
	Exacerbation of chronic obstructive pulmonary disease	0	3
Urological (1)	Urinary tract bleeding	0	1
Cancer (13)	Hepatocellular carcinoma	0	1
	Pancreatic cancer	0	5
	Brain metastases of unknown origin	0	1
	Workup for possible malignancy	0	4
	Neoplastic pleural effusion	1	0
	Generalized carcinomatosis—tension ascites	0	1
Neurological (19)	Epileptic seizures	1	0
	Encephalitis	0	2
	Aphasia	0	1
	Inability to stand	0	3
	Drug-induced coma	0	1
	Fall	1	0
	Refusal to eat, dehydration	2	6
	Self-poisoning	0	1
	Psychotic episode	1	0
Endocrine (3)	Diabetic ketoacidosis	0	2
	Drug-induced hypothyroidism	0	1
Other (17)	Hematoma of the left lower limb	0	2
	Muscle weakness of the upper and lower extremities	1	1
	Acute renal failure	3	4
	Rhabdomyolysis	1	0
	Hypoalbuminemia—edema anasarca	0	1
	Hyponatremia	1	3

* The patients admitted due to anemia were elderly patients admitted in order to receive blood transfusion, with no bleeding-related anemia. Due to their increased PADUA score, they received thromboprophylaxis. ** The patient with ITP was admitted due to low platelets in the context of an infection. He responded rapidly to corticosteroids, but as his hospitalization continued, his platelets rose above 50.000/μL and his PADUA score was increased. He also received thromboprophylaxis.

Table S2. Frequency of possible risk factors for DVT in patients taking antithrombotic therapy. Unfortunately, due to the rarity of the thrombotic incidents, no factor could be statistically associated with thrombosis. DOAC: direct oral anticoagulant.

Risk Factor	Patients (Total nr = 346)
**Thrombosis history**	-
NO	271 (78.3%)

YES	74 (21.4%)
Missing	1 (0.3%)
**If yes, thrombosis type**	-
Other arterial thrombosis	9 (2.6%)
Stroke	28 (8.1%)
Myocardial infarction	24 (6.9%)
DVT	5 (1.4%)
Superficial venous thrombosis	2 (0.6%)
Pulmonary embolism	9 (2.6%)
Splanchnic thrombosis	1 (0.3%)
**Thrombosis history (2)**	-
NO	329 (95.1%)
YES	10 (2.9%)
Missing	7 (2.0%)
**If yes, type of second thrombosis**	-
Other arterial thrombosis	1 (0.3%)
Stroke	1 (0.3%)
Myocardial infarction	5 (1.4%)
Pulmonary embolism	2 (0.6%)
Splanchnic thrombosis	1 (0.3%)
**Bleeding history**	-
NO	321 (92.8%)
YES	22 (6.4%)
Missing	3 (0.9%)
**If yes, bleeding type**	-
Minor	1 (0.3%)
Clinically evident and hemoglobin drop ≥ 2 g/dL within 24 h	5 (1.4%)
Clinically evident and transfusion of ≥2 blood units	2 (0.6%)
Clinically evident and in a critical site (e.g., endocranial retroperitoneal)	3 (0.9%)
That led to discontinuation of treatment	2 (0.6%)
That causes unprogrammed visit to the doctor	1 (0.3%)
Evident bleeding that needs medical assistance	6 (1.7%)
Missing	326 (94.2%)
**Reduced mobility**	-
NO	147 (42.5%)
YES	199 (57.5%)
**Phlebitis/varices (Y/N)**	-
NO	323 (93.4%)
YES	21 (6.1%)
Missing	2 (0.6%)
**Family history of thrombosis**	-
NO	343 (99.1%)
YES	1 (0.3%)
Missing	2 (0.6%)
**Central venous catheter**	-
NO	335 (96.8%)
YES	10 (2.9%)
Missing	1 (0.3%)
**Thrombophilia 1 (known mutation)**	-
Yes	0 (0%)
No	0 (0%)
Missing	346 (100%)
**Thrombophilia 2 (2nd known mutation)**	-

Yes	0 (0%)
No	0 (0%)
Missing	346 (100%)
**Antiphospholipid antibodies**	-
Yes	0 (0%)
No	0 (0%)
Missing	346 (100%)
**Recent surgery**	-
NO	320 (92.5%)
YES	26 (7.5%)
**Heart disease**	-
NO	261 (75.4%)
YES	85 (24.6%)
**Arterial hypertension**	-
NO	314 (90.8%)
YES	32 (9.2%)
**Diabetes mellitus**	-
NO	254 (73.4%)
YES	92 (26.6%)
**Renal insufficiency**	-
NO	295 (85.3%)
YES	51 (14.7%)
**Hepatic failure**	-
NO	344 (99.4%)
YES	2 (0.6%)
**Inflammatory/autoimmune disease**	-
NO	325 (93.9%)
YES	21 (6.1%)
**Thyroid disease**	-
NO	272 (78.6%)
YES	74 (21.4%)
**Dyslipidemia**	-
NO	240 (69.4%)
YES	106 (30.6%)
**Respiratory disease**	-
NO	288 (83.2%)
YES	58 (16.8%)
**DOAC or acenocoumarol use before admission**	-
Apixaban	19 (5.5%)
Dabigatran	1 (0.3%)
Acenocoumarol	4 (1.2%)
Rivaroxaban	4 (1.2%)
None	318 (91.9%)
**Low-molecular-weight heparin use before admission**	-
Fondaparinux	3 (0.9%)
Enoxaparin	17 (4.9%)
Tinzaparin	5 (1.4%)
Bemiparin	2 (0.6%)
None	319 (92.2%)
**Antiplatelet use**	-
NO	284 (82.1%)
YES	56 (16.2%)
None	6 (1.7%)

**Postmenopausal**	-
NO	25 (7.2%)
YES	180 (52.0%)
Missing/non relevant	141 (40.8%)
**Number of deliveries**	-
Mean (SD)	1.51 (0.968)
Median [Min, Max]	2.00 [0, 5.00]
Missing/non relevant	170 (49.1%)
**Complications**	-
Abortion	8 (2.3%)
None	338 (97.7%)
**Complications (2)**	-
Abortion	2 (0.6%)
None	344 (99.4%)
**PADUA score**	-
Mean (SD)	5.55 (1.41)
Median [Min, Max]	5.00 [4.00, 10.0]
